# Single Amino Acid Changes in the Ryanodine Receptor in the Human Population Have Effects *In Vivo* on *Caenorhabditis elegans* Neuro-Muscular Function

**DOI:** 10.3389/fgene.2020.00037

**Published:** 2020-02-26

**Authors:** Brittany Graham, Marie-Anne Shaw, Ian A. Hope

**Affiliations:** ^1^ School of Biology, Faculty of Biological Sciences, University of Leeds, Leeds, United Kingdom; ^2^ Leeds Institute of Medical Research, St James’s University Hospital, Leeds, United Kingdom

**Keywords:** ryanodine receptor, *Caenorhabditis elegans*, *unc-68*, malignant hyperthermia, neuromuscular function, locomotion, central core disease, calcium ions

## Abstract

The ryanodine receptor mediates intracellular calcium ion release with excitation of nerve and muscle cells. Ryanodine receptor missense variants cause a number of myopathologies, such as malignant hyperthermia, and have been linked with various neuropathologies, including Alzheimer’s disease. We characterized the consequences of ryanodine receptor variants *in vivo*. Eight *Caenorhabditis elegans* strains, with ryanodine receptor modifications equivalent to human myopathic *RYR1* variants, were generated by genome editing. In humans, these variants are rare and confer sensitivity to the inhalational anaesthetic halothane when heterozygous. Increased sensitivity to halothane was found in both homozygous and heterozygous *C. elegans*. Close analysis revealed distinct subtle locomotion defects, due to the different single amino acid residue changes, even in the absence of the external triggering agent. Distinct pre- and postsynaptic consequences of the variants were characterized through the responses to cholinergic pharmacological agents. The range of phenotypes reflects the complexity of the regulatory inputs to the ryanodine receptor and the criticality of the calcium ion channel opening properties, in different cell types and with age. Ryanodine receptors with these single amino acid residue changes still function as calcium ion channels, but with altered properties which are likely to have subtle consequences for human carriers of such variants. The long-term consequences of subtly altered calcium ion signalling could be cumulative and may be focussed in the smaller nerve cells rather than the more robust muscle cells. It was important to assess phenotypes *in vivo* to properly appreciate consequences for a whole organism.

## Introduction

The ryanodine receptor (RyR) is a key intracellular calcium ion channel in the sarcoplasmic reticulum (SR) or endoplasmic reticulum (ER) of excitable and non-excitable cells (Zissimopoulos et al., 2006). RyRs mediate Ca^2+^ release from the SR or ER into the cytosol upon excitation of muscle or nerve cells, respectively. Although the three isoforms of RyR are often referred to as skeletal muscle (RyR1), smooth muscle (RyR2), and brain (RyR3) (Marks et al., 1989; Takeshima et al., 1989; Nakai et al., 1990; Ledbetter et al., 1994; Zucchi and Ronca-Testoni, 1997) specific, they are expressed in other tissues. For example, RyR1 is also found in the brain, most prominently in Purkinje cells (Zissimopoulos et al., 2006). RyRs are very large proteins, consisting of four identical monomers of more than 5,000 amino acid residues (Santulli et al., 2018). Described as “mushroom-shaped”, most of the RyR sits in the cytoplasm with a small transmembrane domain forming the pore. The structure of RyRs has been resolved such that the conformational change of the protein upon channel opening has been determined (Efremov et al., 2015; Yan et al., 2015; Zalk et al., 2015). The large cytoplasmic region is key to integration of various critical regulatory inputs.

RyR variants have been associated with a number of pathologies, most notably myopathies, including malignant hyperthermia (MH) (Robinson et al., 2006), age-related decline in skeletal muscle function (Boncompagni et al., 2006; Andersson et al., 2011), and heart arrhythmia (Salvage et al., 2019). Links have also been made, however, to other pathologies, such as neurodegenerative disease (Del Prete et al., 2014). MH is a myopathy typically caused by any one of numerous, typically-dominant, missense variants in RyR1, where exposure to inhalational anaesthetics results in a severe, potentially fatal, reaction due to uncontrolled opening of the Ca^2+^ channel (Carpenter et al., 2009). The genetic prevalence of individuals carrying MH associated variants in the general population is estimated at one in 1,500 individuals (Shaw and Hopkins, 2019) but the total frequency of RyR1 missense variants is much higher. MH susceptibility can be diagnosed through an *in vitro* contracture test (IVCT) using skeletal muscle biopsy tissue (Hopkins et al., 2015). Genetic diagnosis may accompany or be in place of the IVCT should a known MH causing RyR1 variant be present in the family. There are 48 RyR1 variants recognised so far as MH diagnostic by the European MH Group. However, there are over 2500 different missense RyR1 variants in the Genome Aggregation Database (gnomAD) (Karczewski et al., 2019).

MH susceptible (MHS) individuals are generally considered as having no phenotypic changes without anaesthesia (Rosenberg et al., 2015). A correlation was once reported, however, between histopathological appearance and IVCT response for MHS versus MH negative individuals (Mezin et al., 1997). Also, skeletal muscle structural abnormalities have been found in mice with a viable homozygous RyR1 variant knock-in (Yuen et al., 2012). Furthermore, a number of other myopathies associated with RyR1 missense variants, such as Central Core Disease (CCD) and Multiminicore disease (MmD), manifest as muscle weakness, which can range from mild to life threatening (Robinson et al., 2006), and knock-in mice carrying one of the most common CCD RyR1 variants (I4895T) demonstrated reduced muscle strength (Loy et al., 2011). Nevertheless, it is important to fully understand the consequences of missense RyR1 variants *in vivo*, in the absence of a triggering agent, given the frequency of such variants in the human population and the complexity of this channel’s regulatory control.

With the complexity of mammalian systems, the nematode *Caenorhabditis elegans* provides a more convenient and simpler experimental system for study of ryanodine receptor function *in vivo*. *C. elegans* has a single gene, *unc-68*, encoding all the RyR function in the worm, including for both muscle cells and nerve cells. The power of genetic manipulation in *C. elegans* allows for precise missense modifications in the genome to be generated and studied with relative ease. Additionally, *C. elegans* strains carrying *unc-68* modified as per pathological *RYR1* variants, in extrachromosomal arrays, were shown previously to confer MH-related phenotypes of increased sensitivity to IVCT testing agents, caffeine, and the inhalational anaesthetic halothane (Nicoll Baines et al., 2017).

Here, an investigation of *C. elegans* strains carrying missense mutations in the chromosomal copy of *unc-68*, generated using CRISPR-Cas9 genome editing, is reported. Eight mutations corresponding to known or suspected myopathic *RYR1* variants were studied, all affecting residues conserved from *C. elegans* to all three human RyRs. When either homozygous, or heterozygous with a wild type allele, the variants conferred an MH-related phenotype of increased anaesthetic sensitivity. Furthermore, additional phenotypes were seen even in the absence of a triggering agent. These additional phenotypes were observed across a range of ages, from the earliest larval stage to old adults, and were quantified by close analysis of worm locomotion. Assays using drugs to interrogate function at the neuro-muscular synapse revealed distinct phenotypes for the different *unc-68* variants, providing insight into the potential consequences of human RyR1 variants on Ca^2+^ channel function *in vivo*.

## Materials and Methods

### *Caenorhabditis elegans* Culture

All *C. elegans* strains were maintained at 20°C, on 50 mm plates of nematode growth medium (NGM) (3 g NaCl, 17 g Agar, 2.5g Peptone in 975 ml water, autoclaved and cooled to 55°C before addition of 1 ml 1M CaCl_2_, 1 ml 1M MgSO_4_, 1 ml cholesterol (5 mg/ml in ethanol), and 25 ml 1M KPO_4_ (pH 6)) (Stiernagle, 2006). Plates were seeded with 150 µl of an OP50 *Escherichia coli* overnight culture, grown in LB.

### *C. elegans* Strains

*C. elegans* strains were generated by CRISPR-Cas9 genome editing. UL4239 (hR163C) (*le4239*) and UL4285 (hN2342S) (*le4285*) were generated at the University of Leeds from an N2 Bristol strain, obtained from the MRC-LMB, Cambridge. Strains COP1879 (hG341R) (*knu765*), COP1883 (hR2163H) (*knu810*), COP1947 (hR2454H) (*knu825*), COP1944 (hR24548H) (*knu822*), COP1932 (hK3452Q) (*knu810*), and COP1950 (hR4861H) (*knu828*) were generated by NemaMetrix, also through injection of an N2 Bristol strain maintained by NemaMetrix. Due to the different sources of N2 strains used to generate the variant strains, both Leeds N2 and NemaMetrix N2 were assayed. As no significant differences were found, and for simplicity, all references to N2 in this manuscript refer to the N2 strain provided by NemaMetrix due to six of eight RyR1 variant strains originating from this background. The RyR null strain CB540 *(unc-68(e540))* was obtained from the MRC-LMB, Cambridge. The *unc-119* fluorescent reporter strain OH441 (*otls45[unc-119::gfp])* was provided by the Caenorhabditis Genetics Center.

Heterozygous *unc-68* variant/wild type and control individuals were generated by mating. N2 males were mated with OH441 hermaphrodites to generate heterozygous *otls45[unc-119::gfp]/*wild type males, which were then mated with *unc-68* variant strains, CB540 *(unc-68(e540))* and N2. Hermaphrodites carrying the *unc-119::gfp* marker as a result of cross-fertilization were assayed. No males were used in phenotyping assays.

### Synchronisation and Ageing

Young adult, day 10 adult and larval stage one hermaphrodite *C. elegans* were used for phenotyping assays. Larval stage hermaphrodite *C. elegans* were isolated by washing a mixed stage population plate with 1 ml S medium into a microcentrifuge tube. Worms were allowed to settle by gravity for 1 min before pipetting 10 µl of the supernatant to an 8-well slide. By allowing worms to settle, older and larger animals settled more quickly, thus allowing smaller and younger animals to be isolated. L1 animals were discriminated from older stages by size and morphology.

Young adult animals were procured by bleaching mixed stage populations containing gravid adults. The worms in M9 buffer were mixed with 0.3 volumes of a dilute hypochlorite solution, as in household bleach, and 0.2 volumes of 4M NaOH, until only eggs remained. Bleaching kills all post-embryonic stages, while embryos are protected by the egg-shell. Prepared eggs were transferred to seeded plates and allowed to develop. All animals hatched within 14 h of each other. 3.5 days after bleaching, with the presence of a few eggs on the plate, animals were considered young adults. CB540 (*unc-68(e540))* and COP1950 (hR4861H) both took an extra day to reach the young adult stage, as indicated by the onset of egg laying.

Day 10 animals were generated with synchronisation in the same way as for young adults. At the L4 stage, 2 days after synchronisation, animals were washed from 50 mm NGM plates with M9 to 90 mm NGM plates containing 50 µM FUdR (5-fluoro-2’-deoxyuridine) (Sigma-Aldrich) and seeded with 450 µl OP50 *E. coli* overnight culture. FUdR inhibits DNA synthesis, preventing fertilised eggs from developing and hatching, while still allowing adults to lay eggs. There is no somatic cell division in *C. elegans* adults. FUdR was only provided when assay animals are about to mature to avoid developmental abnormalities associated with FUdR treatment. For this reason CB540 and COP1950 were only transferred to FUdR plates on day 3 of adulthood to match the developmental delay. FUdR has been found to extend lifespan in certain instances, as well as induce resistance to stress (Anderson et al., 2016). Here, neither lifespan or stress resistance were assessed, and so it was deemed appropriate to use FUdR to limit progeny hatching.

### Halothane Sensitivity and Swimming Assays

Halothane assays were conducted on individuals of the appropriate age. Well-fed individuals were selected from NGM plates and transferred to 1 ml of S medium, or S medium containing 1 mM, 2.5 mM, or 5 mM halothane. S medium contains 1 litre S Basal (5.85 g NaCl, 1 g K_2_HPO_4_, 6 g KH_2_PO_4_, 1 ml cholesterol (5 mg/ml in ethanol), H_2_O to 1 litre and autoclaved), 10 ml 1 M potassium citrate pH 6, 10 ml trace metals solution (1 Litre stock: 1.86 g Na_2_ EDTA, 0.69 g FeSO_4_•7H_2_O, 0.2 g MnCl_2_•4H_2_O, 0.29 g ZnSO_4_•7H_2_O, 0.025 g CuSO_4_•5H_2_O, H_2_O to 1 litre, autoclaved and stored in the dark), 3 ml 1 M CaCl_2_, 3 ml 1 M MgSO_4_ (Stiernagle, 2006). Body bends were counted after 1 min of exposure. 25 worms were assayed for each strain at each concentration, split into two groups assayed on different days to confirm reproducibility.

Halothane was prepared as a 125 mM stock in DMSO. Just prior to assaying, 500 µl of S medium was added to a well of a 24-well plate, followed by 500 µl of S medium with or without halothane. An individual was immediately transferred into the S medium using a sterile worm pick. This method was used to avoid the halothane/DMSO mixture damaging the plastic of the plate. Immediate onset of the assay is important due to the volatility of halothane.

### L1 Swimming Assays

10 µl of S medium containing *C. elegans* L1s was pipetted into each well of an 8-well microscope slide. *C. elegans* transferred to the slide were allowed 1 min to acclimate. One L1 animal was identified per well of the 8-well slide and body bends were counted for that individual. 32 animals were assayed per strain, across two different days.

### Quantification of Crawling Gait

Animals were grown to the indicated age and kept well fed, on NGM plates. One unseeded 90 mm 25 ml NGM plate at 20°C was used for each strain. Plate temperature is important as it affects the animals’ locomotion. A 1 mm scale was placed on the NGM surface and a picture captured, to set the scale in post recording analysis for each plate, as slight differences in plate volume will affect the scale. 20–30 animals per strain, of the appropriate age, were then picked to the plate and recorded crawling freely for 1 min at 25 fps.

Crawling videos were recorded using a multi-worm tracker system set up by the Cohen group at the University of Leeds. This system used a Navitar telemetric lens and a Ximea xiQ USB camera to capture video of worms crawling on an NGM plate, with illumination by a PolyTec LED red light ring. Videos were recorded at 25 fps using StreamPix 7 (version 7.2.1). Scales were calculated for each video using ImageJ software (Schneider et al., 2012), and videos were analysed subsequently using TierpsyTracker 1.4.0 software (Javer et al., 2018). Analysis examples, available online (https://github.com/aexbrown/tierpsy_tools) were used to work with the timeseries data directly by reading the “features” files from TierpsyTracker 1.4.0 in MATLAB. Primary wavelength, maximum amplitude, midbody crawling frequency, and body length were extracted, providing a compiled dataset of all worms in each tracked frame. Frequency values were converted to positive as dorsal and ventral distinctions were not considered significant. For each animal tracked up to 1,500 data points were recorded, resulting in up to 45,000 data points for each parameter.

### Cholinergic Pharmacology Assays

Levamisole and aldicarb assays were conducted on young adult animals. Aldicarb sensitivity assays were conducted as previously described (Oh et al., 2017; Oh and Kim, 2017). 50 mm NGM plates containing 1 mM aldicarb, from 100 mM aldicarb (Insight Biotechnology) in 70% ethanol, were allowed to dry for at least one day. This concentration of aldicarb induces paralysis after a convenient length of time, allowing different levels of resistance to be readily distinguished. Plates were stored at 4°C and used within 2 weeks of pouring. Plates were allowed to warm to room temperature overnight before use. Using forceps, a 16 mm diameter copper ring was dipped into 70% ethanol and passed through a flame for ~10 seconds. The ring was then placed immediately onto one side of an aldicarb-containing plate and held still with forceps until it had cooled, becoming slightly embedded into the agar plate. A second copper ring was added to the plate in the same way, allowing two assays to be conducted at once. Once the agar had cooled, 10 µl of an OP50 *E. coli* overnight culture was pipetted to the centre of each ring. The combination of food and copper ring is used to corral worms and limit individuals crawling off plates during the assay. 20–30 animals per strain were placed into the ring and observed every 10 min for the first 2 h and at 30 min intervals for the following 2 h. Around 80–100 worms per strain were used, across a minimum of three repeats.

For the levamisole assays, a 2 M stock of levamisole (Sigma) in M9 buffer was used to prepare solutions of 100 µM, 1 mM, 10 mM, and 100 mM in M9 buffer. 10–15 individuals were picked into 1 ml of M9, with or without levamisole, in a well of a 24 well plate. Individuals were observed every 10 min for 2 h and locomotion behaviours were documented. Movement behaviours were then categorised and coded for ease of representation.

### Quantification and Statistical Analysis

Statistical parameters are reported in the figures and corresponding figure legends. None of the data were removed from statistical analysis as outliers. All statistical analysis was performed in GraphPad Prism.

Comparisons of thrashing rate and halothane sensitivity between genotypes in each condition assayed are expressed as 10–90 percentile box and whisker plots. Differences were identified as ns, not significant, or significant to *P < 0.05 or **P < 0.005 using one-way ANOVA. Comparisons between heterozygotes and homozygotes with *unc-68* variant, null or wild type and comparison between homozygotes of the same genotype at different ages were conducted using Sidak’s multiple comparison test for comparison of pre-selected pairs. Comparisons between *unc-*68 variant strains and wild type or the *unc-68* null mutant were conducted using Tukey’s multiple comparison test.

Crawling parameters are represented as scatterplots, for two of the three crawling parameters, with both vertical and horizontal error bars (standard error) for young adult and old adult. Error bars which are smaller than the height of the data point are not drawn. Statistical analysis of differences between the strains is not presented here due to the large number of repeats resulting in significance in most comparisons. This type I error can occur in very large data sets where small differences can turn out to be statistically significant. However, when the small differences are considered in the context of the practical significance, they are not large enough to be considered meaningful. Kymograms for single individuals were generated using the Tierpsy Tracker 1.4.0 software and MatLab. Colour bars are limited from 1 to -1. Strains with more green have wider bend angles, while strains with deeper reds and blues have more acute bend angles. Solid black vertical lines represent frames where the individual worm’s skeleton could not be tracked by the software.

Survival fractions in aldicarb were displayed as a Kaplan-Meier survival curve in GraphPad Prism. Individual strain’s survival curves were compared to wild type (or the *unc-68* null mutant for COP1950 (hR4861H)) using a Gehan-Breslow-Wilcoxon test.

## Results

### *RYR1* Variant Versions of *unc-68* Conferred an MH-Related Halothane Sensitivity Phenotype

Eight *RYR1* missense variants associated with skeletal muscle pathology in the human population, and which affect single amino acid residues conserved in the *C. elegans* ryanodine receptor UNC-68, were selected. Point mutations were introduced into the genomic copy of *unc-68*, in the N2 wild type strain, through CRISPR-Cas9 genome editing such that the only change to the amino acid sequence of the gene product corresponded to human RyR1 variants R163C, G341R, R2163H, N2342S, R2454H, R2458H, K3452Q, and R4861H (**Table 1**). Additional modifications were made around the target codon to prevent further genome editing but these would not change the ryanodine receptor amino acid sequence (**Supplementary Table 1**).

**Table 1:** Amino acid alignment of human RyR1 and *C. elegans* UNC-68 in the regions of the studied variants, and the *C. elegans* strain expressing the variant UNC-68.

Human RyR1 variant (*C. elegans* variant)		Alignment	*C. elegans* strain
R163C (R169C)	RyR1	ASKQRSEGEKV**R**VGDDIILVS	UL4239
	UNC-68	ASKQRSEGEKV**R**VGDDVILVS	
G341R (G350R)	RyR1	PPEIKY**G**ESLCFVQHVASGLW	COP1879
	UNC-68	NATIRY**G**ETNAFIQHVKTQLW	
R2163H (R2246H)	RyR1	LLECLGQI**R**SLLIVQMGPQEE	COP1883
	UNC-68	FLVYLIQI**R**ELLTVQFEHTEE	
N2342S (N2441S)	RyR1	DFLRFAVFV**N**GESVEENANVV	UL4285
	UNC-68	DFLRFCVWI**N**GENVEENANLV	
R2454H (R2560H)	RyR1	AGKGEALRI**R**AILRSLVPLED	COP1947
	UNC-68	AGKGDSLRA**R**AILRSLISLDD	
R2458H (R2564H)	RyR1	AGKGEALRIRAIL**R**SLVPLED	COP1944
	UNC-68	AGKGDSLRARAIL**R**SLISLDD	
K3452Q (K3675Q)	RyR1	IYWSKSHNF**K**REEQNFVVQNE	COP1932
	UNC-68	RIWSQSQHF**K**REELNYVAQFE	
R4861H (R5021H)	RyR1	VVVYLYTVVAFNFF**R**KFY-NK	COP1950
	UNC-68	VVVYLYTVIAFNFF**R**KFYVQE	

The residue changed in the variants is in bold and underlined. The UNC-68 residue numbering is based on isoform a.

**Figure 1:**
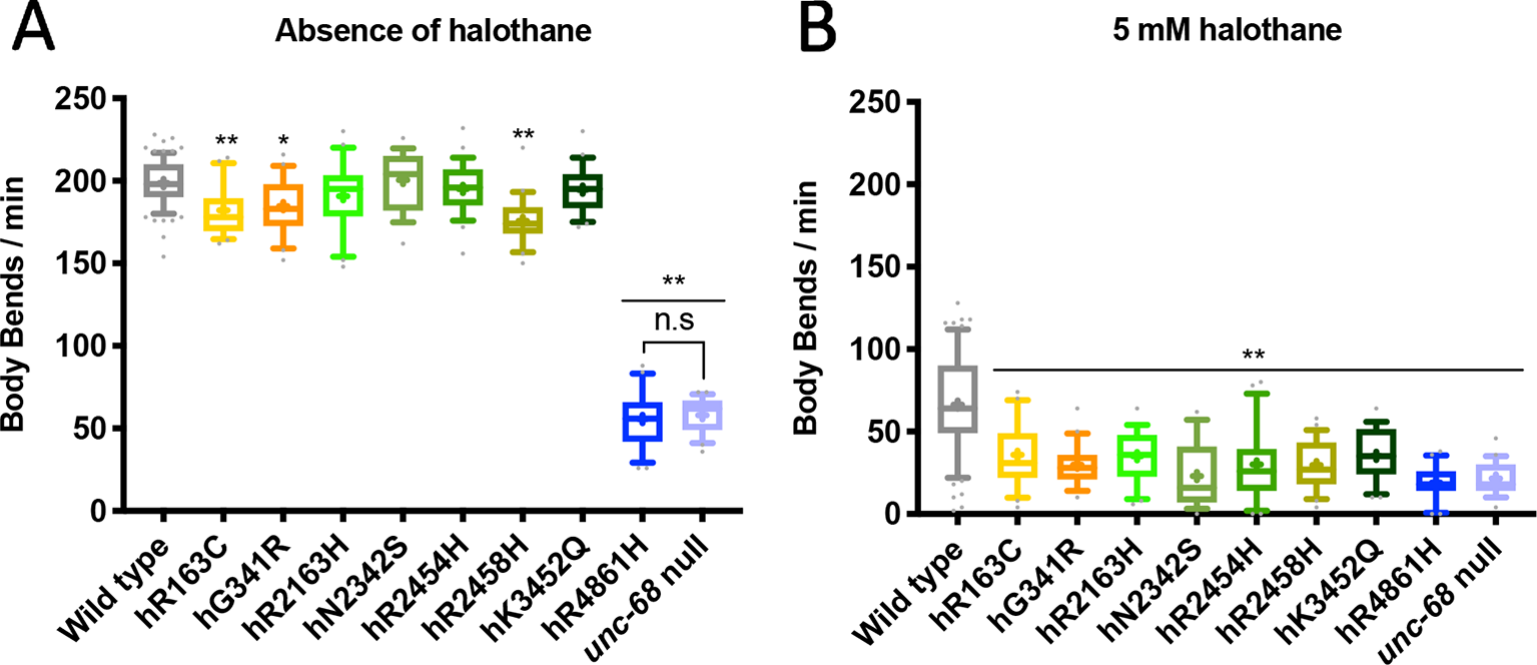
Ryanodine receptor (RyR) variants confer a malignant hyperthermia (MH)-related phenotype of hypersensitivity to halothane. Thrashing rate in S medium, in body bends per minute, for RyR variant strains, labelled by the human variant they correspond to, in the absence of **(A)** and presence of **(B)** 5 mM halothane / 4% DMSO. 25 individual young adults were examined per strain. Boxes indicate the median and interquartile range, with whiskers to the 10–90 percentile, outliers as dots, and + to indicate the mean. Significance is between variant strains and the N2 wild type, apart from where indicated to the CB540 *unc-68(e540*) null mutant. *P < 0.05, ** *P* < 0.005, n.s, not significant (one-way ANOVA, with Tukey’s multiple comparison test). Colouring corresponds to variant location in the protein. Yellow/orange variants are located within the N-terminal domain (NTD), green in the helical domain (HD), and blue in the transmembrane domain (TMD). Gray represents wild type and lilac the null mutant.

Eggs prepared from populations of each *C. elegans* strain were allowed to develop and halothane sensitivity was assessed when animals reached young adulthood. The rate of locomotion in liquid was recorded at a range of halothane concentrations. A progressive decline in rate of thrashing was seen for all strains in increasing concentrations of halothane, including the wild type strain N2. However, all *unc-68* mutant strains demonstrated a much slower rate of thrashing in 5 mM halothane compared to the wild type (**Figure 1**). For seven of the eight strains expressing *RYR1* based *unc-68* variants, thrashing rate in the absence of halothane was, in contrast, close to or indistinguishable from that of the wild type. This increased sensitivity to halothane, conferred by these single amino acid changes in the ryanodine receptor, reflects the anaesthetic sensitivity of *RYR1*-associated malignant hyperthermia. Increased halothane sensitivity was seen previously upon expression of missense *unc-68* variants, but from a high-copy number, extrachromosomal location (Nicoll Baines et al., 2017), rather than the natural genomic location assayed here. Expression from the endogenous *unc-68* location is expected to provide the normal amount of a gene product rather than the higher amounts potentially produced from the extrachromosomal location. Differing halothane sensitivities were conferred by the different ryanodine receptor variants as revealed with assays of locomotion at lower halothane concentrations (**Supplementary Figure 1**). This variation parallels the varying MH phenotypes for different *RYR1* genotypes (Carpenter et al., 2009).

One *unc-68* variant, that for RyR1 hR4861H, conferred a much-reduced thrashing rate even in the absence of halothane. This variant strain, COP1950, was indistinguishable from the *unc-68(e540)* null mutant strain, CB540, in this assay. This single amino acid change appeared to totally inactivate the ryanodine receptor.

### Aging Increases Sensitivity to Halothane for the Wild Type but Not for RyR1 Variant Strains

Links between ageing and RyR1 variants have been made previously (Boncompagni et al., 2006; Andersson et al., 2011; Meissner, 2017; Nicoll Baines et al., 2017). Age is also identified as a factor of relevance to manifestation of RyR1-related myopathies (Litman et al., 2018). The median lifespan for wildtype *C. elegans* is 15 days into adulthood (Gems and Riddle, 2000), however, strains transgenic for variant *unc-68*s, in extrachromosomal arrays, had shorter lifespans than wild type (Nicoll Baines et al., 2017). Halothane sensitivity was therefore re-evaluated for the genome edited strains at day 10 of adulthood, when the worms were considered to be aged, for comparison to young adults at day 1 of adulthood. Synchronised animals, grown from prepared eggs, were maintained on NGM plates containing 5-fluoro-2’-deoxyuridine from the L4 stage to prevent progeny hatching. This allowed assay of aged animals without confusion with subsequent generations.

**Figure 2:**
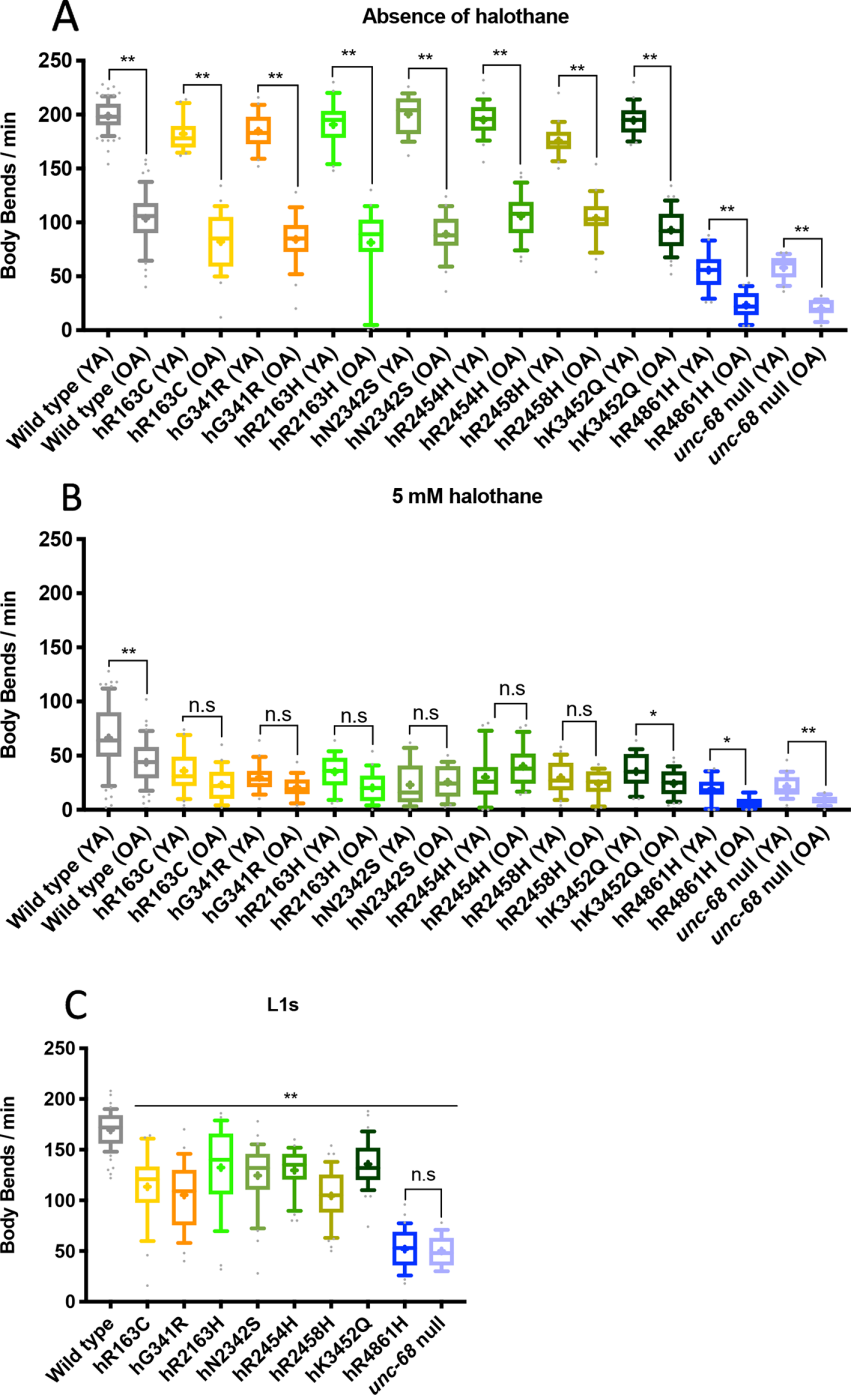
Locomotion is differentially affected across the lifespan by ryanodine receptor (RyR) variants. Thrashing rate in S medium, in body bends per minute, for RyR variant strains, labelled by the human variant they correspond to, along with the N2 wild type and the CB540 *unc-68(e540*) null mutant. Comparisons are between young adult (YA) and old adult (OA) in the absence **(A)** and presence **(B)** of 5 mM halothane or between strains as L1s in the absence of halothane **(C)**. 25 individuals were examined per strain. Boxes indicate the median and interquartile range, with whiskers to the 10–90 percentile, outliers as dots, and + to indicate the mean. Significance is between ages of each variant strain **(A, B)** or to the wild type **(C)**. *P < 0.05, **P < 0.005, n.s, not significant [one-way ANOVA, with Sidak’s multiple comparison test **(A**, **B)**, and Tukey’s multiple comparison test **(C)**].

Individuals at day 10 of adulthood were assayed for halothane sensitivity as before. Thrashing rate for old adults was approximately half that seen in young adults, in the absence of halothane, in all strains, reflecting a general reduction of locomotion as the animals age (**Figure 2A**). For most of the *unc-68* variant strains, the thrashing rate of old adults in the presence of 5 mM halothane was not significantly less than that of young adults, although it was for the wild type (**Figure 2B**). That the thrashing rate of aged adults in the presence of halothane was not reduced further while the thrashing rate in the absence of halothane was, suggests that in most of the variant strains, with a modified RyR, halothane is having less of an effect in aged animals than in younger animals. The halothane could have reduced the thrashing rate of these old adults further, as seen for the *unc-68* null mutant and the hR4861H *unc-68* variant strain, which behaves as a null mutant. The effect of halothane appears as strong in old adults as in young adults in the presence of wild type *unc-68*, although the variant strains remain less motile in the presence of halothane than the wild type. Curiously, hK3452Q, the only functional variant for which the strain showed a significant further reduction of locomotion of old adult versus young adult in the presence of halothane, corresponds to that associated with Late Onset Axial Myopathy, a condition that manifests in older human individuals. However, the effect is small and this could be just coincidental. Nevertheless, the relative locomotion of variant strains as compared to the wild type and the *unc-68* null mutant in the presence and absence of halothane for old adults (**Supplementary Figure 2**) was very similar to that for young adults.

### RyR Variant Strain L1s Have Reduced Thrashing Rate

Given the reduced effect of halothane on aged adults as compared to young adults, in the presence of RyR variants, the effects of RyR1 variants on larvae was assessed. It was surmised that the animals might gradually adapt to the presence of a defective RyR as they age and, if so, the consequences of RyR variants could be more marked in younger worms. Larval stage one, L1, is the earliest stage where *C. elegans* are hatched from the egg and are freely moving. L1s were collected from mixed stage plates and frequency of body bends was counted for single individuals.

At the L1 stage, in the absence of halothane, all RyR variants caused a markedly reduced thrashing rate compared to N2 wild type (*P* < 0.005) (**Figure 2C**). The hR4861H variant caused the most reduced L1 locomotion, once again matching that of the *unc-68* null mutant. However, the effect on locomotion due to the other RyR variants was more substantial for L1s than for adults. The reduction in L1 thrashing rate in liquid in the absence of halothane was significant for all the variant strains, not just some.

### RyR Variant Strain Crawling Is Distinguishable From Wild Type

**Figure 3:**
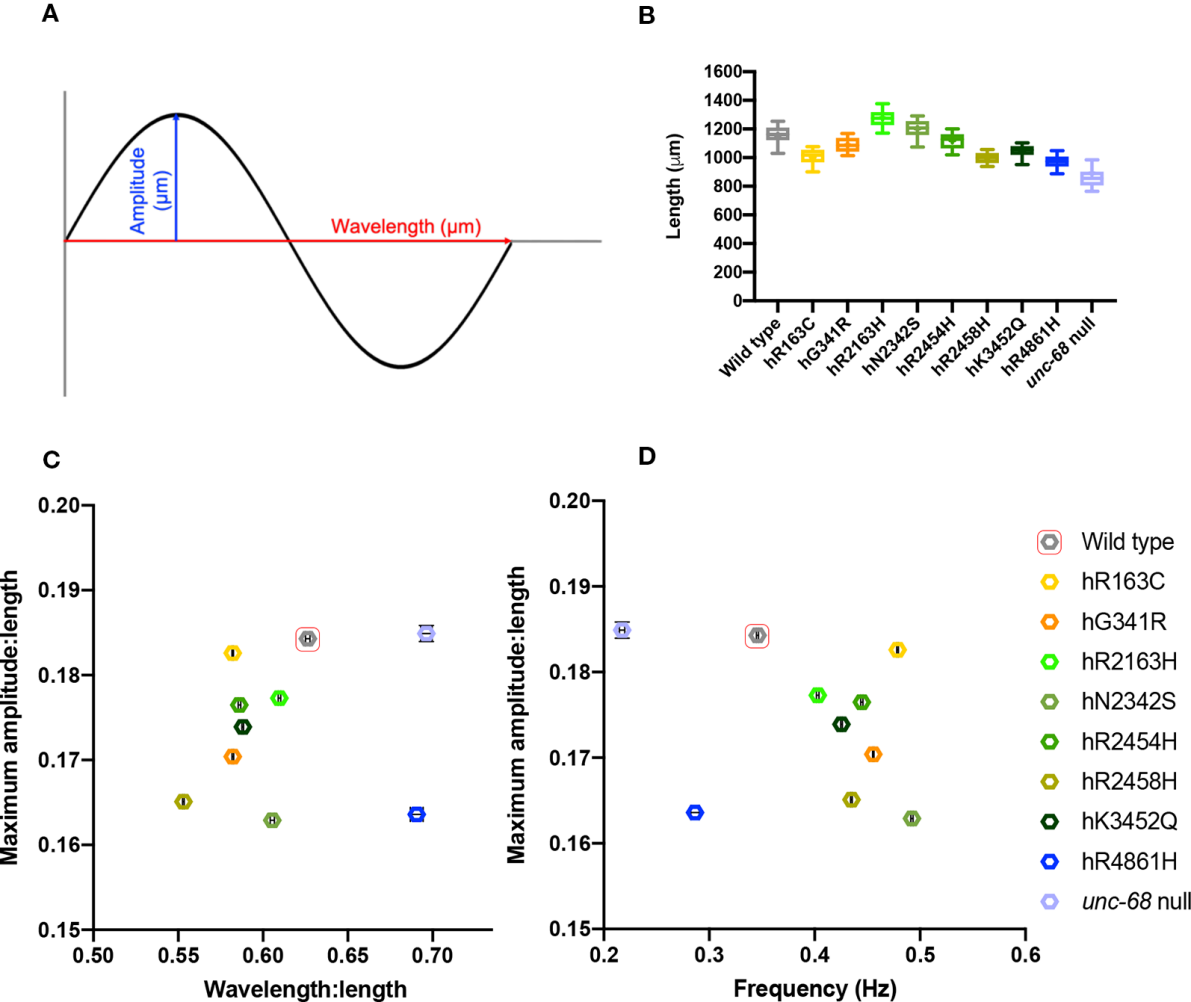
Young adult crawling differences due to ryanodine receptor (RyR) variants. Amplitudes and wavelengths of the crawling wave form **(A)** were extracted from 1 min long, 25 frames per second, video recordings of 20–30 individual young adults, along with worm length and wave frequency. Worm length **(B)**, maximum amplitude versus wavelength **(C)**, and maximum amplitude versus frequency **(D)** for RyR variant strains, labelled by the human variant they correspond to, along with the N2 wild type and the CB540 *unc-68(e540*) null mutant, were compared. Maximum amplitude and wavelength were normalized for worm length. Boxes indicate the median and interquartile range, with whiskers to the 10–90 percentile, outliers as dots, and + to indicate the mean **(B)**. Mean and SEM are indicated, although SEM is very small for frequency and for normalized maximum amplitude and wavelength **(C, D)**.

To characterize locomotion in the RyR variant *C. elegans* strains more closely, crawling on agar was examined. Crawling is a slower movement than thrashing, and is restricted to a flat surface, creating a sinusoidal wave in the animal’s dorsal-ventral plane which can be more easily measured and quantified. Young adults were videoed and the maximum amplitude, midbody frequency, primary wavelength and body length, during crawling, were extracted (**Figure 3**, **Supplementary Table 2**). Computer analysis of the videos involved 1,500 data points for each individual, and 20 to 30 individuals were analysed per strain, providing robust quantification of locomotion parameters.

Mean lengths of age synchronised worms varied between the different strains, suggesting RyR variants affect growth and/or development of the animals (**Figure 3B**). The *unc-68* null mutant has delayed development, matching that for the hR4861H variant strain. However, the other RyR variants also affect development, but more subtly. Most of the RyR variant strains had a mean length shorter than wild type, although the strains for variants hR2163H and hN2342s were slightly longer. Due to the differences, wavelength, and amplitude of the locomotion were normalized with respect to worm length.

The maximum amplitude of a worm’s crawling motion is the distance between the midline and the peaks of the locomotion sine wave. Once corrected for differences in length, the different strains had only subtle differences in maximum amplitude. The mean maximum amplitude for all strains was between 0.16 to 0.19 times the mean length for that strain. The wild type and the *unc-68* null mutant had the largest mean maximum amplitude, with respect to length, and were found to be indistinct from each other upon statistical analysis, (*P* = 0.9991, One-way ANOVA with Tukey’s test). All of the RyR1 variant strains had smaller amplitude:length ratios and were significantly different to both wild type and the null mutant. Of note, is the large difference in mean maximum amplitude:length ratio seen for the *unc-68* null mutant and the hR4861H variant strain, suggesting that this variant is not actually a null mutant, as suggested from the liquid assays, and some functionality remains.

The primary wavelength of the crawling is the direct distance between the peaks of the sine wave, again assessed with respect to worm length. Wavelength was found to range from 0.55x to 0.7x the length of the worm. For this parameter, the hR4861H variant strain and the *unc-68* null mutant were statistically indistinguishable (*P* = 0.2341, One-way ANOVA with Tukey’s test), and these strains had the largest wavelength:length ratios (0.691x (*se* = 0.004) and 0.696x (*se* = 0.003) the length respectively). The wild type had a larger wavelength:length ratio than the other variant strains at 0.626x (*se* = 0.001) the mean length. Wavelength:length ratio was very similar for most of the other RyR variant strains ranging from 0.582x to 0.609x the length of the worm, except for the hR2458H variant strain, which had a much shorter wavelength (0.553x (*se* = 0.0005) the length).

While the differences for amplitude and wavelength, with respect to worm length, were subtle, when plotted together the variant strains sit quite separately from the wild type and the *unc-68* null mutant (**Figure 3C**). All the variant strains have a shorter amplitude and/or shorter wavelength than the wild type and *unc-68* null mutant. The combination of shorter amplitude and shorter wavelength, as in most of the RyR1 variant strains, results in a tighter bend angle during crawling.

Midbody crawling frequency, expressed in waves per second, is a measure of the time it takes for one complete body bend to propagate from head to tail. All RyR variant strains had a higher mean crawling frequency than the wild type (0.49 Hz (*se* = 0.001 Hz) to 0.40 Hz (*se* = 0.001 Hz) versus 0.35 Hz (*se* = 0.001 Hz)), except for that for hR4861H. The mean crawling frequency for the hR4861H variant (0.29 Hz (*se* = 0.004 Hz)) was lower than for the wild type but higher than for the *unc-68* null mutant (0.22 Hz (*se* = 0.003 Hz)). While absence of RyR function reduced frequency for both thrashing in liquid and crawling on agar, most of the RyR1-based single amino acid changes increased crawling frequency. Again, the differences between the strains is clear when amplitude and frequency are plotted against each other (**Figure 3D**).

**Figure 4:**
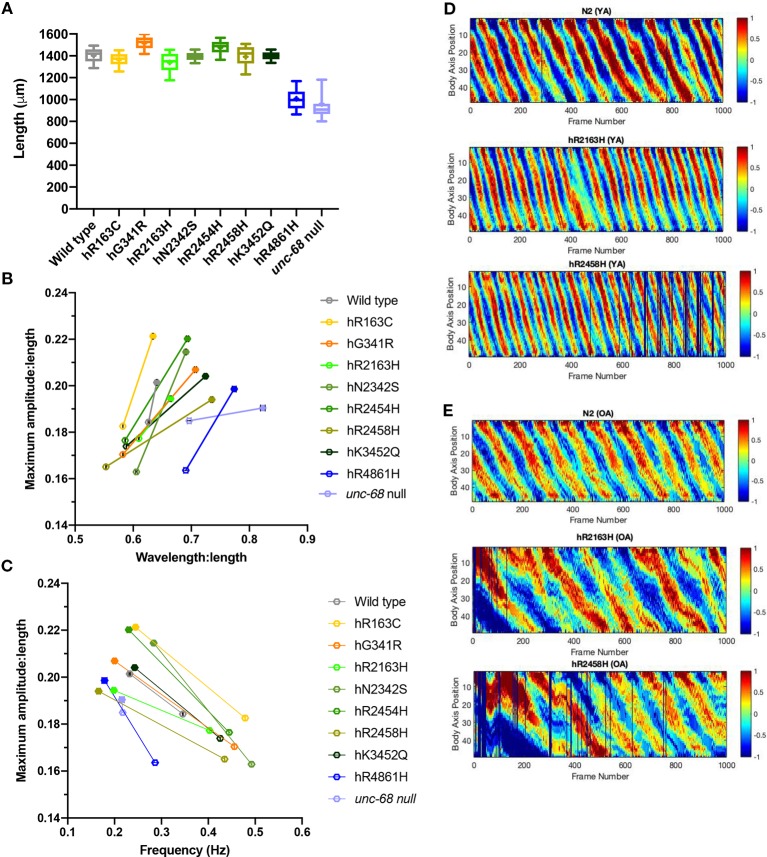
Effect of ryanodine receptor (RyR) variants on crawling changes with ageing. Crawling amplitudes, wavelengths and frequencies, and worm lengths, were extracted from 1 min long, 25 frames per second, video recordings of 20–30 individual 10-day old adults. Worm length **(A)**, maximum amplitude versus wavelength **(B)** and maximum amplitude versus frequency **(C)** for RyR variant strains, labelled by the human variant they correspond to, along with the N2 wild type and the CB540 *unc-68(e540*) null mutant, were compared. Maximum amplitude and wavelength were normalized for worm length. Boxes indicate the median and interquartile range, with whiskers to the 10–90 percentile, outliers as dots, and + to indicate the mean **(A)**. Mean and SEM are indicated, although SEM is very small for frequency and for normalized maximum amplitude and wavelength **(B, C)**. The change in maximum amplitude and primary wavelength from young adult (hollow symbols) to old adult (solid symbols), is indicated by a straight line **(B, C)**. Representative kymograms are presented for the N2 wild type and for hR2163H and hR2458H RyR variant strains for **(D)** young adults (YA) and **(E)** old adults (OA). Colour indicates degree of dorsal/ventral curvature (red/blue high curvature, green no curvature) at different positions along the body axis (0 anterior to 50 posterior) for each frame of the video recording. Individual frames where parameters could not be extracted are black.

The crawling parameters provide an insight into the effects of these single amino acid changes on RyR function *in vivo* and the consequences for Ca^2+^ dynamics. Those effects can be readily appreciated from comparison of the kymograms (see later, **Figure 4D**) which emphasize the differences in the crawling behaviour. The higher frequencies than wild type for most of the RyR variant strains might be expected because their smaller wavelength and amplitude means their movement is less pronounced.

### Crawling Parameters Reveal Different RyR Variants Have Distinct Ageing Consequences

Careful analysis of crawling on agar surfaces revealed locomotion differences between RyR variant strains in young adults which were not apparent for thrashing in liquid. Therefore, crawling parameters were also determined for old adults, at day 10 of adulthood (**Table 2**, **Figure 4**, **Supplementary Table 2**).

**Table 2:** Mean percentage change from young adult to old adult in maximum amplitude and primary wavelength, both corrected for worm length, and in midbody frequency.

Strain	Maximum Amplitude:length	Primary Wavelength:length	Midbody Frequency
Wild type	+2%	+9%	-33%
hR163C	+9%	+21%	-49%
hG341R	+21%	+21%	-56%
hR2163H	+9%	+10%	-51%
hN2342S	+14%	+32%	-42%
hR2454H	+18%	+25%	-48%
hR2458H	+33%	+18%	-62%
hK3452Q	+23%	+17%	-43%
hR4861H	+12%	+21%	-38%
*unc-68* null	+18%	+3%	-1%

Percentages are for ryanodine receptor (RyR) variant strains, listed according to the human variant they correspond to, along with the N2 wild type and the CB540 unc-68(e540) null mutant.

Worm lengths of old adults also exhibited differences between strains suggesting that RyR variants affect adult growth as well as earlier development (**Figure 4A**). The *unc-68* null mutant and the hR4861H variant strain are much shorter than the other strains as old adults, reflecting little growth between young and old adult. The body length variation between the other RyR variant strains, and for the other variant strains compared to the wild type, was not as marked in the old adults as in the young adults.

Both the amplitude and wavelength of locomotion, both with respect to body length, increased in all strains from young to old adult (**Table 2**, **Figure 4B**). The smallest change was for the wild type, with only a 2% increase in amplitude and 9% increase in wavelength. The amplitude increase of the *unc-68* null mutant was substantial at 18% but the wavelength increase was even smaller than the wild type at just 3%. In comparison, for all RyR variant strains, the amplitude increase with age was larger than for the wild type ranging from 9% to 33%, and the wavelength increase was larger than for both the wild type and *unc-68* null mutant at 10 to 32%.

Midbody crawling frequency decreased for all strains with age (**Table 2**, **Figure 4C**), the aged adults moving much more slowly, with the exception of virtually no change for the *unc-68* null mutant. For N2 and variants hR163C, hN2342S, hR2454H and hK3452Q, crawling frequency remained ≥ 0.23 Hz. For variants hG341R, hR2163H, hR2458H, and hR4861H and the *unc-68* null mutant, crawling frequency was ≤ 0.22 Hz.

The changes in crawling wavelength, amplitude and frequency with age, seen for all strains, means that body bending is shallower and slower in older individuals. These differences are immediately apparent in the kymograms (**Figures 4D, E**). Aged animals do not contract body wall muscles to the same extent and as quickly as young adults. This effect of ageing is more dramatic in the RyR variant strains than in the wild type.

### RyR Variants Have Distinct Presynaptic Effects

RyRs are important for nerve cell, as well as muscle cell, function. For example, RyRs have been shown to have a specific role in the release of the neurotransmitter acetylcholine (ACh) at mouse neuromuscular junctions (NMJs) (Khuzakhmetova et al., 2014). In *C. elegans*, UNC-68 has also been shown to have a presynaptic as well as a postsynaptic role (Liu et al., 2005). The variant amino acid changes could have effects on RyR function in nerve cells or muscle cells or both. Therefore, the consequences of RyR variants, in our genome edited strains, with respect to cholinergic neurotransmission at *C. elegans* NMJs were assessed.

Application of aldicarb and levamisole has been used to distinguish between pre- and post-synaptic effects of *C. elegans* mutations (Rand, 2007). Aldicarb is an inhibitor of *C. elegans* acetylcholinesterase (AChE) and prevents the breakdown of ACh to cause a build-up of synaptic ACh, the excitatory transmitter at NMJs, resulting in hypercontracted paralysis. Mutations resulting in an excess of neurotransmitter release from the presynaptic cell result in a more rapid build-up of ACh, and thus more rapid paralysis, in the presence of aldicarb (Rand, 2007). Conversely mutations disrupting ACh release lead to aldicarb resistance and slower paralysis. Contributions from postsynaptic effects have been distinguished using levamisole (Lewis et al., 1980). Levamisole is a cholinergic agonist, binding specifically to levamisole-sensitive nicotinic ACh receptors (L-nAChRs), present at NMJs. The activation of the postsynaptic membrane by levamisole results in muscle hypercontraction, which is typically followed by relaxation and death. Mutations with postsynaptic effects can modify sensitivity to levamisole. Assessment of strains for both aldicarb and levamisole resistance could reveal details of the consequences of the RyR variants for cholinergic neurotransmission.

**Figure 5:**
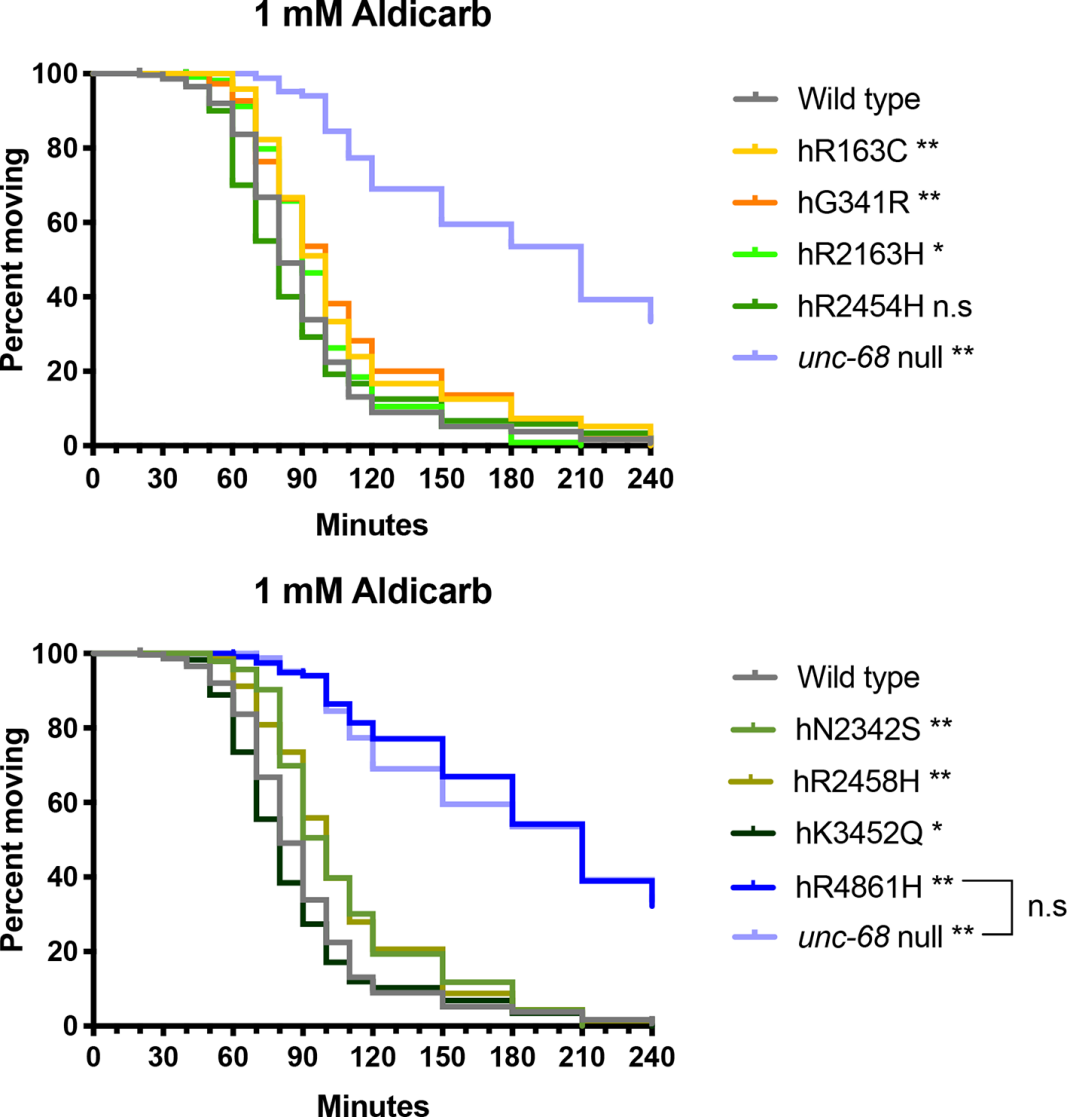
Ryanodine receptor (RyR) variants alter time to paralysis in 1 mM aldicarb. Kaplan-Meier survival curves representing the percentage of individuals still moving with time in 1 mM aldicarb. Data for the RyR variant strains, labelled by the human variant they correspond to, along with the N2 wild type and the CB540 *unc-68(e540*) null mutant are distributed across two graphs for clarity. Comparison to the wild type, and of the hR4861H variant strain to the *unc-68* null mutant (square bracket), to assess if curves were significantly different, employed the Gehan-Breslow-Wilcoxon test; *P < 0.05, *P < 0.005, n.s, not significant.

The strains for RyR1 variants hR2454H and hK3452Q responded very similarly to aldicarb and appeared slightly more sensitive than the N2 wild type (**Figure 5**). Although all three strains showed the same median time to paralysis of 80 min, this could reflect a lack of resolution due to sampling intervals. Analysis of the Kaplan-Meier curves revealed the difference from N2 was only just statistically significant for the hK3452Q variant, and not at all for the hR2454H variant. In contrast, the six other RyR variant strains exhibited resistance to aldicarb in comparison to N2. The strain for variant hR2163H had a median time to paralysis of 90 min, while four other variant strains, hR163C, hG341R, hN2342S, and hR2458H exhibited further resistance with 100 min median paralysis times. The strain for the hR4861H variant showed the strongest resistance, once again behaving like the *unc-68* null mutant, with both having median paralysis times of 210 min.

Initial levamisole sensitivity assays used a conventional concentration of 1 mM (Qian et al., 2008). Movement of individuals from the different strains, in liquid, was noted at 10 min intervals for 2 h (**Table 3**). In the absence of levamisole, individuals of each strain remained swimming rapidly throughout. After an hour, swimming became less smooth for short intervals, but did continue. The N2 wild type worms all became straight and paralysed by 1 mM levamisole within 20 min. The *unc-68* null mutant and the hG341R, hR2163H, and hN2342S variant strains responded to levamisole to the same degree or very similarly to the wild type. The hR2163H mutation appeared to cause a slight increase in sensitivity, with paralysis by 10 min. The hN2342S mutation appeared to cause a slight decrease in sensitivity with some individuals showing weak, occasional twitching, curling, folding, shivering or head waving, which we refer to as “attenuated movement”, from the onset of paralysis at 20 min, until complete paralysis of all individuals after 100 min. The hR4861H mutation caused a more substantial decrease in levamisole sensitivity, responding quite differently in this assay from the *unc-68* null mutant. Attenuated movement continued in all hR4861H strain individuals until 30 min and in some individuals from 60 min onwards, as if becoming partially refractile to the levamisole.

**Table 3:** Response of ryanodine receptor (RyR) variant strains, identified by the human variant they correspond to, and of the N2 wild type and the CB540 *unc-68(e540*) null mutant, to 1 mM levamisole in M9 buffer over 2 h.

	Time (min)												
	0	10	20	30	40	50	60	70	80	90	100	110	120
Wild type	2	3	0	0	0	0	0	0	0	0	0	0	0
hR163C	2/3	3/4	4	4	4	4	4	3/4	3	3	3	3	3
hG341R	1/2	2/3	0	0	0	0	0	0	0	0	0	0	0
hR2163H	2/3	0	0	0	0	0	0	0	0	0	0	0	0
hN2342S	1/2	3	0/3	0/3	0/3	0/3	0/3	0/3	0/3	0/3	0/3	0	0
hR2454H	2	3	3	3	3/4	3/4	3/4	3	3	3	0/3	0/3	0/3
hR2458H	1/2	3	3	4	4	4	4	4	4	4	4	4	4
hK3452Q	1/2	3	3	4	4	4	4	4	4	4	3/4	3/4	3/4
hR4861H	2/3	3	3	3	0	0	0/3	0/3	0/3	0/3	3	3	0/3
*unc-68* null	2/3	2/3	0	0	0	0	0	0	0	0	0	0	0

No movement 0, No movement with attenuated movement 0/3, Swimming 1, Uncoordinated swimming 2, Attenuated movement 3, Kinking 4.

More strikingly, several RyR variant strains, those for hR163C, hR2454H, hR2458H, and hK3452Q, exhibited a novel “kinking” phenotype in 1 mM levamisole (Video available online at https://www.dropbox.com/s/uout8m64ly3gm1a/17-46-28.000.avi?dl=0). These worms exhibited intermittent sharp bending in both directions at kinks between straight and rigid sections, in varying positions down the anterior/posterior axis. While some individuals of the hR2454H strain did eventually show complete paralysis from 100 min, no individuals of the hR163C, hR2458H, and hK3452Q strains showed complete paralysis across the 2 h of observation. There was some variation between the variants in the time of switching to the kinking phenotype from a less specific locomotion defect, and eventually back again. This phenotype appeared weakest for hR2454H, with no time point when all individuals exhibited kinking, and strongest for hR2458H, with kinking retained by all individuals until the end of observation.

To explore if the kinking phenotype could be induced in the other strains, the response to higher and lower concentrations of levamisole was investigated. This provided further support for the increased sensitivity of the hR2163H strain as compared to the wild type, with all individuals rapidly paralysed even at 100 μM levamisole. Also, partial recovery from complete paralysis at higher levamisole concentrations was observed for several of the strains, including the wild type, with the shift to and from complete paralysis becoming earlier with increasing levamisole concentrations. At 100 mM levamisole, complete paralysis could be induced eventually in all strains. However, while the kinking phenotype was seen at higher levamisole concentrations for the RyR variant strains which had expressed this phenotype in 1 mM levamisole, the kinking phenotype was never observed for the wild type, the *unc-68* null mutant or any of the other RyR variant strains.

In liquid, the response of the *unc-68* null mutant to levamisole appears very similar to wild type. The partial levamisole resistance described previously for *unc-68* null mutants on an agar surface (Lewis et al., 1980), may be attributed to the slower worm body flexing or greater force requirement, making intracellular Ca^2+^ release more critical for muscle contraction than in worms in liquid. The novel kinking phenotype of four of the RyR variant strains and the greater levamisole sensitivity of the hR2163H strain, in liquid, are quite distinct from the phenotype of the *unc-68* null mutant and therefore reflect a gain of an activity in an aspect of RyR function as a result of the single amino acid changes. Gain of function phenotypes typically show dominance over the wild type and malignant hyperthermia susceptibility is mostly a dominant condition (Monnier et al., 2002).

### RyR Variants Conferred a Locomotion Defect That Was Dominant to Wild Type

Many *RYR*-related pathologies, including most instances of MH, exhibit genetic dominance and the condition is expressed in heterozygous individuals (Monnier et al., 2002). Therefore, heterozygotes, with the modified *unc-68* alleles over the wild type allele, were tested for the MH-related phenotype of increased halothane sensitivity. Homozygous RyR variant strain hermaphrodites were mated with *unc-68* wild type males. Heterozygous, young adult, cross-progeny hermaphrodites were distinguished from the self-progeny and transferred individually into liquid medium with or without halothane.

**Figure 6:**
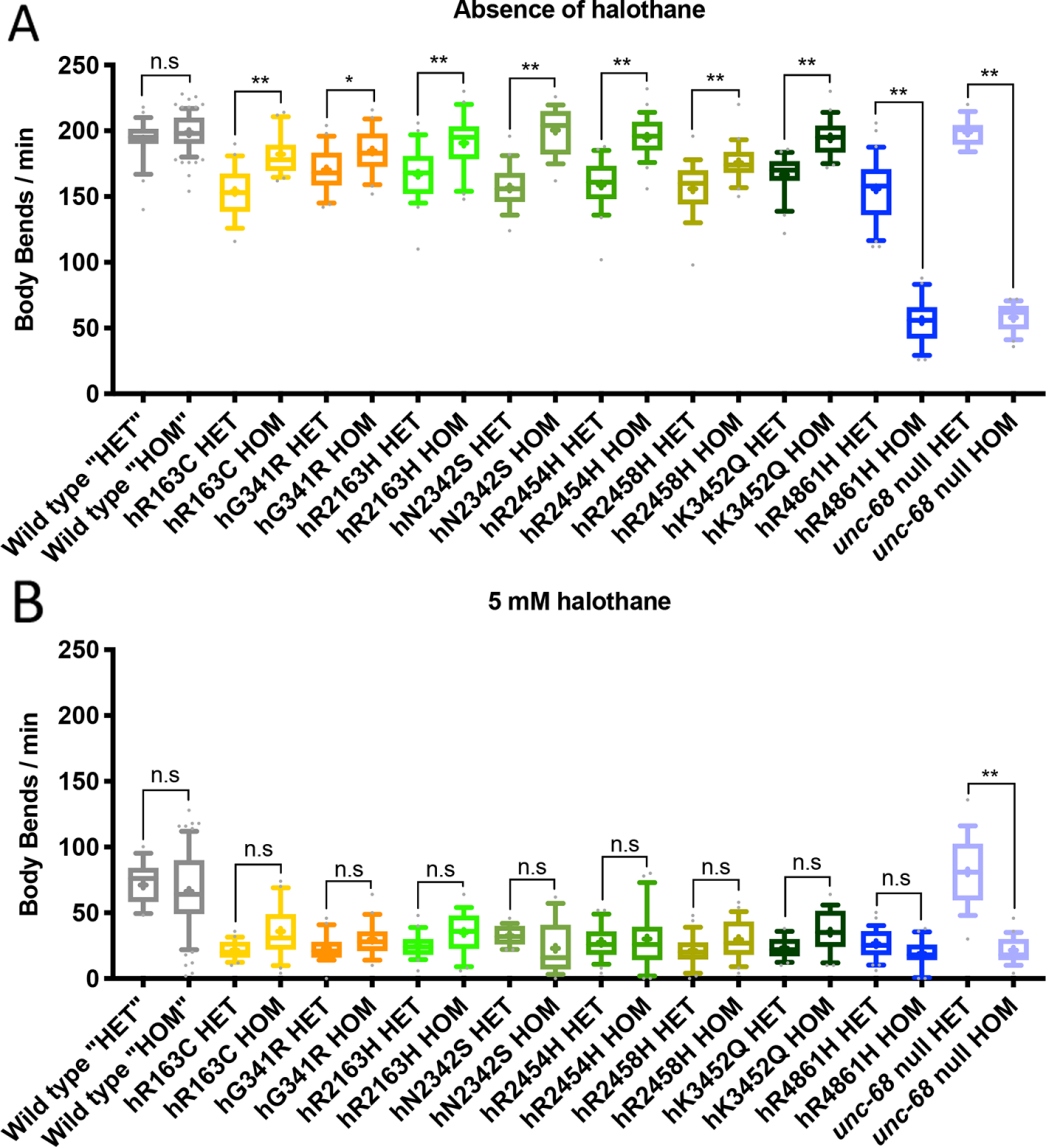
Locomotion effects in heterozygous ryanodine receptor (RyR) variant individuals. Thrashing rate in S medium, in body bends per minute, for 25 individuals, in the absence of **(A)** and presence of **(B)** 5 mM halothane. Those expressing RyR variants are identified by the human variant they correspond to, and were either heterozygous (HET), with the modified *unc-68* over a wild type *unc-68* introduced by mating, or homozygous (HOM) for the modified *unc-68*. Corresponding N2 wild type and *unc-68(e540*) null mutant individuals were generated and assayed in the same way, in parallel, for direct comparison, although the wild type “heterozygotes” (“HET”) and “homozygotes” (“HOM”) are fully wild type for *unc-68*. Boxes indicate the median and interquartile range, with whiskers to the 10–90 percentile, outliers as dots, and + to indicate the mean. Significance is between heterozygotes and homozygotes. *P < 0.05, **P < 0.005, n.s, not significant (one-way ANOVA, with Sidak’s multiple comparison test).

MH is typically described as asymptomatic without the application of inhalational anaesthetics (Rosenberg et al., 2015). However, the heterozygotes for all the RyR variants had slower locomotion in liquid than the wild type control even in the absence of halothane (**Figure 6**, **Supplementary Figure 3**). The single amino acid changes in UNC-68 conferred an effect on neuro-muscular function even in normal conditions and in the presence of wild type versions of the protein.

The *unc-68* null phenotype is considered recessive to wild type (Maryon et al., 1996). Similarly, here, when heterozygotes are generated in the same way as for the RyR variant strains, but from the null mutant, the rate of locomotion in liquid was indistinguishable from the wild type control and much faster than the homozygous null (**Figure 6**). One copy of wild type *unc-68* is sufficient to rescue the null phenotype. In contrast, a wild type copy of *unc-68* did not fully rescue the locomotion defect of the hR4861H *unc-68* variant. Even though the hR4861H variant strain behaved in liquid like the null mutant when homozygous (**Figure 6**), this allele is clearly not a null. The aberrant hR4861H UNC-68 is expressed and can interfere with the function of the wild type protein, as is the case in the human condition associated with this missense RyR1 variant.

Remarkably, with the exception of hR4861H, the heterozygotes had a stronger locomotion defect than the RyR variant homozygotes. Some RyR variants which failed to show a locomotion defect, in the absence of halothane, as homozygotes, now showed such a defect as heterozygotes. The presence of a wild type UNC-68 enhances the consequences of expressing a variant UNC-68 for neuromuscular function.

When exposed to halothane all RyR1 variant heterozygotes exhibited halothane hypersensitivity compared to the N2 wild type (**Supplementary Figure 3**). These results are consistent with the typically dominant inheritance pattern of *RYR1* associated myopathies like MH. However, the rate of locomotion of the heterozygotes, in liquid in the presence of halothane, was indistinguishable from that for the homozygotes for the RyR variant strains. The presence of the wild type UNC-68 did not enhance the halothane sensitivity due to the variant UNC-68.

## Discussion

The consequences recorded previously (Nicoll Baines et al., 2017) for mutations in the *C. elegans* RyR gene, equivalent to human myopathic variations, have now been observed with mutations in the endogenous chromosomal location instead of in an extrachromosomal array. These mutations increase sensitivity to halothane, a reflection of the human condition. Genome editing of *unc-68*, rather than using a transgene, means the modified RyR expression level and distribution reflects that of the wild type. Normal expression of the modified RyRs has revealed additional significant phenotypes. Effects of the RyR mutations were observed even without the external triggering agent, i.e., in the absence of halothane. These included effects of age, subtle posture changes while crawling over a support surface and consequences of the presence of a wild type RyR.

First stage larvae of all the RyR variant strains exhibited a strongly reduced rate of bending while thrashing in liquid, in comparison to the wild type. In contrast, a significant reduction in rate of locomotion in liquid, was small and only observed for some of the *unc-68* mutations in young and old adults. *C. elegans’* postembryonic development from L1 to young adult, involves both the nervous system, with five of the eight motor neuron classes generated at the end of L1, and the muscular system, with 14 of the 95 adult body wall muscles being added (Sulston and Horvitz, 1977). During the L1 stage, one class of ventral cord motor neurons undergoes complete synaptic reorganisation in terms of their synaptic pattern in relation to dorsal and ventral body wall muscle. With much of the neuromuscular system undergoing development through and after L1, mutations affecting Ca^2+^ levels in these cells may have a larger impact at this stage, or the simpler neuromuscular system of L1s may make this stage more sensitive to perturbation in RyR function. Alternatively, the physiology of the worm could adapt gradually through the lifespan specifically to the subtle defects in RyR function due to the *unc-68* mutations, progressively improving locomotion with age.

In depth analysis of crawling on an agar surface revealed that all strains expressing RyR variants are phenotypically distinct from both the wild type and the *unc-68* null mutant in this environment too. Differences in crawling amplitude and wavelength are small for both young and old adults but presumably reflect differences in RyR Ca^2+^ channel properties, with consequences for Ca^2+^ dynamics during muscle or nerve excitation. Such consequences would alter Ca^2+^ levels achieved and maintained in different cellular compartments. Examination of the degree of change reveals the increase in crawling amplitude and wavelength with age is significantly more marked for the variant strains than wild type and may reflect reductions in strength and/or increase in duration of muscle contraction. This could arise from the different Ca^2+^ dynamics. However, Ca^2+^ leakage through RyRs has been suggested to lead to progressive cellular damage. Blocking “leaky” RyRs in old mice resulted in decreased reactive oxygen species (ROS) and increased muscle-specific force and exercise capacity (Andersson et al., 2011). A self-reinforcing cycle of RyR leakage and mitochondrial ROS production may lead to age-related muscle dysfunction, as demonstrated in *RYR1* variant knock-in mice where older variant mice had reduced maximal tension which was preventable with antioxidant supplementation (Durham et al., 2008). Some Ca^2+^ leakage occurs through the wild type RyR. Increased Ca^2+^ leakage or flow, due to *unc-68* missense mutations could lead to more damage with age.

The difference in worm length between strains could be due to RyR variants affecting feeding through disturbing either the neural control or the muscle contraction of pharyngeal pumping. Mutations which resulted in reduced pharyngeal pumping or inefficient pharyngeal pumping were found previously to lead to shorter worms (Morck and Pilon, 2006). Links have been made between the pharynx in *C. elegans* and cardiac pathology associated with human RYR2 variants (Fischer et al., 2017).

Thrashing defects due to RyR variants were obvious in the heterozygotes, with a wild type copy of *unc-68*, even if not apparent in the homozygote. Previously, in the absence of the triggering agent, no effect on locomotion due to RyR variants, expressed in *C. elegans* from mixed variant/wild type extrachromosomal arrays as a model of the heterozygous state, had been discerned (Nicoll Baines et al., 2017). Presumably, UNC-68 expression levels are critical for a variant UNC-68 to cause an obviously altered phenotype upon interaction with the wild type channel, in unchallenged conditions, and expression from the endogenous locus provides that.

Although the homozygous hR4861H variant strain behaved very similarly to the *unc-68* null mutant, thrashing of the heterozygote, in the presence and absence of halothane, matched that of other variant strains. R4861 lies within the transmembrane, pore-forming, domain of RyR1 (Yan et al., 2015), the only example amongst the variants studied here, and may be the most critical to channel function. RyR is a tetramer, however, and the hR4861H variant RyR subunit, like the others, must be stably expressed, and incorporated into the majority of RyR channels in the heterozygotes. Such incorporation must be sufficient to interfere with, but not prevent, functioning of the channel. The operation of the small proportion of channels in the heterozygotes which would be composed of only wild type subunits is either insufficient or also interfered with.

Strikingly, for all variants investigated, apart from hR4861H which appears to block channel function, the homozygote moves better in liquid than the heterozygote. This suggests that if all subunits are distorted in the same way by an amino acid residue replacement, the channel works more like the wild type, in most cases, than if it is a mixture of normal and variant subunits. A possible explanation could involve the amino acid changes impacting upon the different aspects of RyR function (Witherspoon and Meilleur, 2016). For example, in a homozygote, an amino acid change might interfere both with channel closure and activated Ca^2+^ flow rate, while in the heterozygote the presence of wild type channel subunits allows a better Ca^2+^ flow rate but the channel still closes slowly. As a consequence, failure to close the channel promptly results in even more Ca^2+^ leakage from the sarcoplasmic reticulum in the heterozygote than in the homozygote. Pathological *RYR* variants in the human population are present at low frequency and so are almost always heterozygous. Nevertheless, our data suggest subtle functional alteration should be expected for heterozygous individuals even in the absence of a trigger.

RyRs function in all cells but are particularly critical in excitable cells. The *C. elegans* locomotion phenotypes could be due to the missense mutations modifying RyR function within the nervous system, affecting neurotransmitter release onto muscle cells, and/or within muscle cells, with a more direct impact on muscle cell contraction.

The altered responses to the AChE inhibitor aldicarb suggests that RyR variants have presynaptic consequences in *C. elegans*. RyR misfunction has been linked to neurodegeneration, including Alzheimer’s disease (Del Prete et al., 2014), and *C. elegans* has been used extensively as an experimental system with which to investigate the role of amyloid in such neurodegeneration (Griffin et al., 2017). Six of the variants studied appear to reduce the amount of neurotransmitter release to cause aldicarb resistance, while the other two variants, hR2454H and hK3452Q, have slightly the opposite, if any, effect. In excited nerve cells, RyRs are stimulated to release Ca^2+^ from the endoplasmic reticulum (ER) into the cytosol by calcium-induced calcium release (CICR), increasing the concentration of Ca^2+^ in the axoplasm and thus increasing the release of neurotransmitters (Narita et al., 2000; Khuzakhmetova et al., 2014). In the RyR variant strains which show resistance to aldicarb, presumably the Ca^2+^ level is lower, upon nerve cell excitation, thus reducing ACh release at the synapse. RyR channel opening could be delayed or channel closing could be premature, reducing Ca^2+^ release from the ER through the RyR. Alternatively, increased Ca^2+^ leakage through the channel could reduce Ca^2+^ levels in the ER, resulting in less Ca^2+^ being available for release upon excitation, and/or lower resting Ca^2+^ levels in the cytosol limiting the increase achieved upon excitation. Of course, different variants could affect any of these steps, and the opposite considerations would apply for RyR variants which confer aldicarb sensitivity.

An altered response of a mutant to levamisole, which activates L-type nicotinic ACh receptors on the postsynaptic muscle cell membrane, is taken to suggest an effect of the mutation within muscle cells (Rand, 2007). However, while several RyR variant strains showed subtle changes in sensitivity to levamisole, the most striking observation was the strong kinking phenotype for four variants. The kinking phenotype is reported for mutations in *C. elegans* genes of the unc (uncoordinated) class typically involved in neural function, and has been interpreted as a conflict between forward and backward locomotion commands. Perhaps the disturbance of Ca^2+^ control due to these RyR variant results in instability across the nervous system which manifests as random excitation of inhibitory motor neurons and consequent temporary relaxation of a few body wall muscles otherwise contracted due to the levamisole.

Notably, strains for the different RyR variants did not show consistency of responses across the different assay types. The two variants that conferred increased aldicarb sensitivity (hR2454H and hK3452Q) did both confer the kinking phenotype in response to levamisole and were amongst the set showing the greatest change in crawling parameters with age. Three of the four variants which failed to show the levamisole induced kinking phenotype (hG341R, hR2163H, and hR4861H) did also have an increased aldicarb resistance and showed little change in crawling parameters with age. The former and the latter phenotypes may equate to increased and decreased RyR Ca^2+^ flow/leakage, respectively. However, the other three variants (hR163C, hN2342S, and hR2458H) do not conform to this pattern and are even distinct from each other. Different consequences of the various RyR mutations may arise from the distinct ways in which Ca^2+^ release from the ER could be affected and/or from disruption of any of the numerous regulatory inputs into RyR function (Kushnir et al., 2018). Amino acid changes could even affect regulation of other proteins by the RyR, as suggested for the hR163C mutation (Estève et al., 2010). Alternatively, the inconsistencies between the RyR variants could reflect differences in physiologies between nerve cell types or between nerve cells and muscle cells. Nevertheless, this variation echoes the variation in human conditions associated with *RyR* missense variants.

Despite the differences, all RyR variants tested in *C. elegans* conferred increased sensitivity to halothane, reduced the rate of body bends in liquid and showed genetic dominance. Most ryanodinopathies are similarly expressed in heterozygous individuals, exhibiting genetic dominance (Robinson et al., 2006). However, the presence of an altered *C. elegans* phenotype due to these RyR variants in the absence of an external trigger may have implications for human carriers of apparently benign as well as pathogenic *RYR* variants. Although most of the missense variants in the three *RYR* genes in the human genome are present at low frequency in the human population there are thousands of different variants affecting residues conserved between RyR1, RyR2, and RyR3 (Lek et al., 2016). That conservation suggests that those residues are likely to be of some functional value. RyRs can clearly tolerate many amino acid changes without serious consequences for routine Ca^2+^ channel operation, but which may well have more subtle effects. The large amount of individual variation in the human population means that comparatively small differences in RyR function may not be readily detectable. Assessment of RyR variants within an intact organism, like *C. elegans*, is important for a proper understanding of the effects, including cumulative, long-term consequences of ageing.

## Data Availability Statement

All datasets generated for this study are included in the article/**Supplementary Material**.

## Author Contributions

Conceptualisation and Methodology: IH. Investigation and Formal Analysis: BG. Resources: IH. Writing – Original Draft and Visualisation: IH and BG. Writing – Reviewing and Editing: IH, M-AS and BG. Supervision: IH and M-AS. Project Administration: IH. Funding Acquisition: IH and M-AS.

## Funding

This research was supported by a Biotechnology and Biological Sciences Research Council grant (BB/M00032X/1) and by the BBSRC White Rose Doctoral Training Programme (BB/M011151/1). Some strains were provided by the CGC, which is funded by NIH Office of Research Infrastructure Programs (P40 OD010440).

## Conflict of Interest

The authors declare that the research was conducted in the absence of any commercial or financial relationships that could be construed as a potential conflict of interest.
