# Supplementary material for: Single Amino Acid Changes in the Ryanodine Receptor in the Human Population Have Effects In Vivo on Caenorhabditis elegans Neuro-Muscular Function
Source: Front Genet. 2020 Feb 26;11:37. doi: 10.3389/fgene.2020.00037 (PMC7054344; doi:10.3389/fgene.2020.00037)
Supplement: Supplementary file 1 [file Image_1.pdf]

Supplementary Material

Supplementary Figure 1

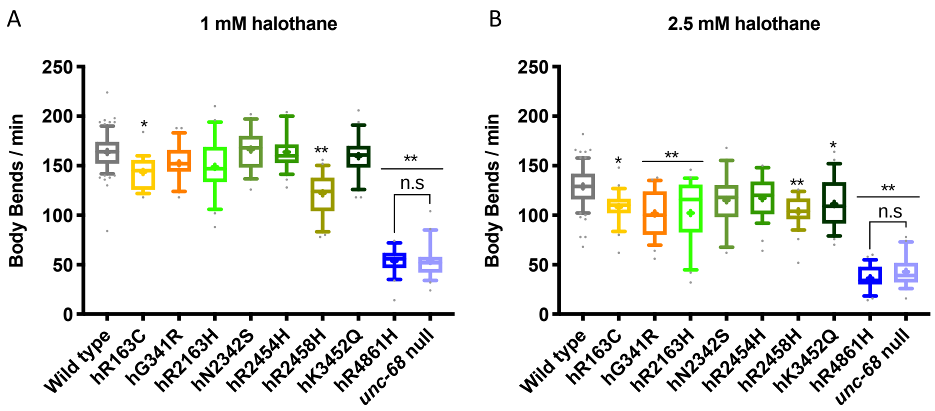

Supplementary Figure 1. RyR variant strains show differing sensitivities to lower concentrations of halothane. Thrashing rate in S medium, in body bends per minute, for RyR variant strains, labelled by the human variant they correspond to, in 1 mM (A) and 2.5 mM (B) halothane. 25 individuals were examined per strain. Boxes indicate the median and interquartile range, with whiskers to the 10-90 percentile, outliers as dots, and + to indicate the mean. Significance is between variant strains and the N2 wild type, apart from where indicated to the CB540 unc-68(e540) null mutant. * P<0.05, ** P<0.005, n.s = not significant (one-way ANOVA, with Tukey's multiple comparison test).
