# Supplementary material for: Single Amino Acid Changes in the Ryanodine Receptor in the Human Population Have Effects In Vivo on Caenorhabditis elegans Neuro-Muscular Function
Source: Front Genet. 2020 Feb 26;11:37. doi: 10.3389/fgene.2020.00037 (PMC7054344; doi:10.3389/fgene.2020.00037)
Supplement: Supplementary file 2 [file Image_2.pdf]

Supplementary Material

Supplementary Figure 2

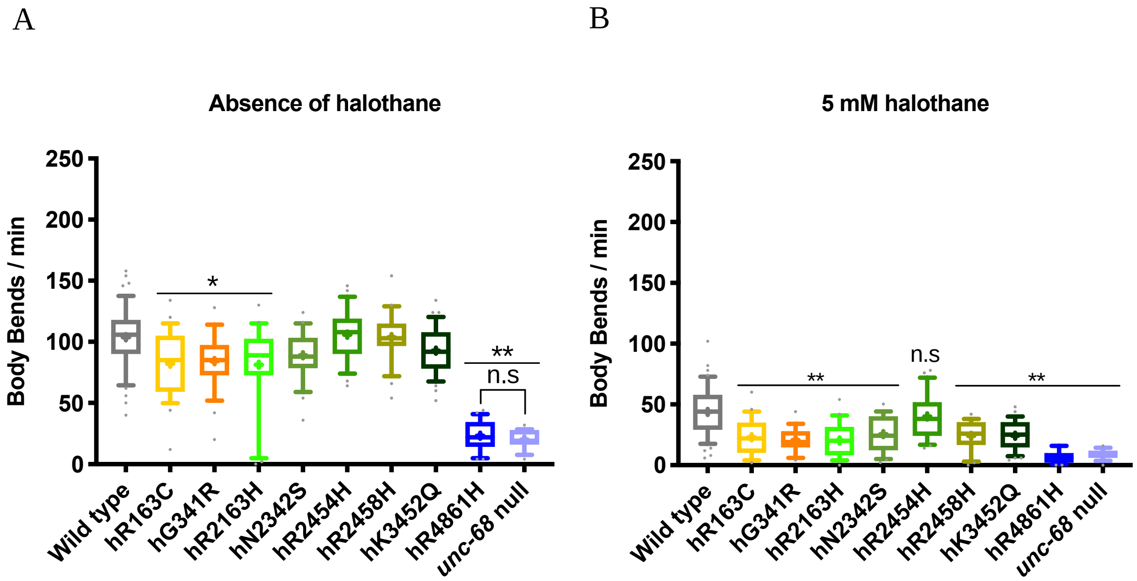

Supplementary Figure 2. Comparison of the locomotion of old adults of RyR variant strains.

Thrashing rate in S medium, in body bends per minute, for old adults of RyR variant strains, labelled by the human variant they correspond to, in the absence of (A) and presence of (B) 5 mM halothane. 25 individual old adults were examined per strain. Boxes indicate the median and interquartile range, with whiskers to the 10-90 percentile, outliers as dots, and + to indicate the mean. Significance is between variant strains and the N2 wild type, apart from where indicated to the CB540 *unc-68(e540)* null mutant. * *P*<0.05, ** *P*<0.005, n.s = not significant (one-way ANOVA, with Tukey's multiple comparison test).
