# Supplementary material for: Single Amino Acid Changes in the Ryanodine Receptor in the Human Population Have Effects In Vivo on Caenorhabditis elegans Neuro-Muscular Function
Source: Front Genet. 2020 Feb 26;11:37. doi: 10.3389/fgene.2020.00037 (PMC7054344; doi:10.3389/fgene.2020.00037)
Supplement: Supplementary file 3 [file Image_3.pdf]

Supplementary Material

Supplementary Figure 3

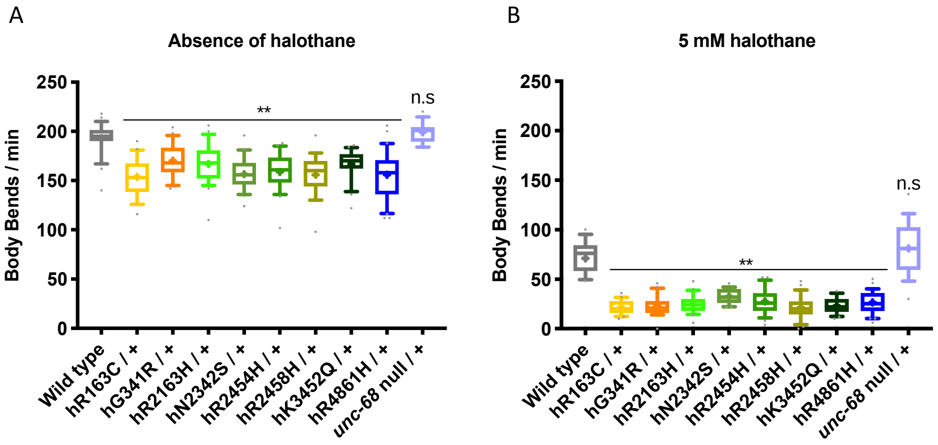

Supplementary Figure 3. Heterozygous RyR variant individuals have reduced locomotion and are more sensitive to halothane as compared to wild type. Thrashing rate in S medium, in body bends per minute, for 25 individual young adults, in the absence of (A) and presence of (B) 5 mM halothane. Those expressing RyR variants are identified by the human variant they correspond to, and were heterozygous (/+), with the modified *unc-68* over a wild type *unc-68* introduced by mating. Corresponding N2 wild type and *unc-68(e540*) null mutant individuals were generated and assayed in the same way, in parallel, for direct comparison. Boxes indicate the median and interquartile range, with whiskers to the 10-90 percentile, outliers as dots, and + to indicate the mean. Significance is between variant heterozygotes or the *unc-68* null and wild type. * $P<0.05$, ** $P<0.005$, n.s = not significant (one-way ANOVA, with Sidak's multiple comparison test). All RyR heterozygotes were indistinguishable from one another ($P>0.05$ one-way ANOVA, Tukey's multiple comparisons), yet were all distinct from wild type ($P<0.005$ one-way ANOVA, Tukey's multiple comparisons), both in the presence and absence of halothane. The *unc-68* null heterozygote was indistinguishable from wild type both with and without halothane ($P=0.389$ and $P=0.995$ one-way ANOVA, Tukey's multiple comparisons).
