# Supplementary material for: Single Amino Acid Changes in the Ryanodine Receptor in the Human Population Have Effects In Vivo on Caenorhabditis elegans Neuro-Muscular Function
Source: Front Genet. 2020 Feb 26;11:37. doi: 10.3389/fgene.2020.00037 (PMC7054344; doi:10.3389/fgene.2020.00037)
Supplement: Supplementary file 4 [file Table_1.docx]

| **Strain (RyR1 variant)** | **Alignment** | |
| --- | --- | --- |
| UL4239 (hR163C) | WT | GCATCCAAACAAAGATCAGAAGGAGAAAAGGTG**C**GCGTCGGTGATGACGTCATTTTGGTCTCA |
|  | VAR | GCATCCAAACAAAGATCAGAAGGAGAAAAAGTC**T**GTGTCGGTGATGACGTCATTTTGGTCTCA |
|  | WT | A S K Q R S E G E K V  **R**  V G D D V I L V S |
|  | VAR | A S K Q R S E G E K V  **C**  V G D D V I L V S |
| COP1879 (hG341R) | WT | AAATGCTACAATCAGATAT**G**GAGAGACAAATGCTTTTATTCAACACGTGAAAACTCAGCTCTGG |
|  | VAR | CAACGCCAC**C**ATTAGATAT**A**GAGAGACAAATGCTTTTATTCAACACGTGAAAACTCAGCTCTGG |
|  | WT | N A T I R Y  **G**  E T N A F I Q H V K T Q L W |
|  | VAR | N A T I R Y  **R**  E T N A F I Q H V K T Q L W |
| COP1883 (hR2163H) | WT | TTCCTGGTGTACCTCATACAAATCC**G**CGAGCTTCTTACCGTACAATTTGAGCATACTGAAGAG |
|  | VAR | TTCCTGGTGTACCTCATCCAGATTC**A**CGAGCTTCTTACCGTACAATTTGAGCATACTGAAGAG |
|  | WT | F L V Y L I Q I  **R**  E L L T V Q F E H T E E |
|  | VAR | F L V Y L I Q I  **H**  E L L T V Q F E H T E E |
| UL4285 (hN2342S) | WT | GATTTCCTGAGATTCTGTGTCTGGATC**AA**TGGGGAAAACGTGGAAGAAAATGCAAATCTTGTC |
|  | VAR | GATTTCCTGAGATTCTGTGTCTGGATA**TC**TGGGGAAAACGTGGAAGAAAATGCAAATCTTGTC |
|  | WT | D F L R F C V W I  **N**  G E N V E E N A N L V |
|  | VAR | D F L R F C V W I  **S**  G E N V E E N A N L V |
| COP1947 (hR2454H) | WT | CCAATGGCTATACAG *INTRON* GCCGGAAAAGGAGATTCTCTTCGCGCTC**G**TGCTATTCTCAGATCTCTTATTTCACTCGACGATCTTGGTCAGATCTTGGCTCTAAGATTTACAATCCCC |
|  | VAR | CCTATGGCCATCCAA GCTGGTAAGGGTGACTCCCTCCGTGCCC**A**CGCCATCCTCCGTTCCCTCATCTCCCTTGATGACCTCGGACAAATCCTCGCCCTCCGTTTCACCATTCCA |
|  | WT | P M A I Q A G K G D S L R A  **R** A I L R S L I S L D D L G Q I L A L R F T I P |
|  | VAR | P M A I Q A G K G D S L R A  **H** A I L R S L I S L D D L G Q I L A L R F T I P |
| COP1944 (hR2458H) | WT | CCAATGGCTATACAG *INTRON* GCCGGAAAAGGAGATTCTCTTCGCGCTCGTGCTATTCTCA**G**ATCTCTTATTTCACTCGACGATCTTGGTCAGATCTTGGCTCTAAGATTTACAATCCCC |
|  | VAR | CCTATGGCCATCCAA GCTGGTAAGGGTGACTCCCTCCGTGCCCACGCCATCCTCC**G**TTCCCTCATCTCCCTTGATGACCTCGGACAAATCCTCGCCCTCCGTTTCACCATTCCA |
|  | WT | P M A I Q A G K G D S L R A R A I L  **R**  S L I S L D D L G Q I L A L R F T I P |
|  | VAR | P M A I Q A G K G D S L R A R A I L  **H**  S L I S L D D L G Q I L A L R F T I P |
| COP1932 (hK3452Q) | WT | ACGGACGGAGTATATGAAAATGTAGCTGTCATCTTCCGTATTTGGAGTCAAAGTCAACATTTC**A**AACGTGAAGAGCTGAACTATGTGGCTCAATTTGAA |
|  | VAR | ACCGACGGCGTCTACGAGAACGTCGCCGTCATTTTCAGAATCTGGTCCCAATCCCAACACTTC**C**AACGCGAGGAGCTCAACTACGTCGCCCAATTCGAA |
|  | WT | T D G V Y E N V A V I F R I W S Q S Q H F **K** R E E L N Y V A Q F E |
|  | VAR | T D G V Y E N V A V I F R I W S Q S Q H F  **Q** R E E L N Y V A Q F E |
| COP1950 (hR4861H) | WT | ACACTTGTAGTCGTGTATCTCTACACTGTCATCGCGTTCAATTTCTTCC**G**TAAATTCTATGTTCAAGAGGGTGAAGAGGGCGAAGAG |
|  | VAR | ACCCTCGTCGTAGTCTACCTCTATACCGTCATTGCCTTCAACTTTTTCC**A**CAAGTTCTACGTCCAAGAAGGAGAAGAGGGCGAAGAG |
|  | WT | T L V V V Y L Y T V I A F N F F  **R**  K F Y V Q E G E E G E E |
|  | VAR | T L V V V Y L Y T V I A F N F F  **H**  K F Y V Q E G E E G E E |

Supplementary Material

**Supplementary Table 1.** Aligned *unc-68* nucleic acid sequence and encoded UNC-68 amino acid sequence of wild type (WT) and variant (VAR) after CRISPR-Cas9 genome editing, for each *C. elegans strain*. Bold underlined bases and amino acids correspond to the point mutations which change the amino acid sequence. Red underlined residues correspond to all mutations in the variant genomic sequence, including silent mutations introduced to prevent re-editing of the genome during strain generation. The intron (grey) in the wild type has been deleted in the mutant COP1947 (hR2454H) and COP1944 (hR2458H) strains.
