# Supplementary material for: Single Amino Acid Changes in the Ryanodine Receptor in the Human Population Have Effects In Vivo on Caenorhabditis elegans Neuro-Muscular Function
Source: Front Genet. 2020 Feb 26;11:37. doi: 10.3389/fgene.2020.00037 (PMC7054344; doi:10.3389/fgene.2020.00037)
Supplement: Supplementary file 5 [file Table_2.docx]

Supplementary Material

| Young Adults | | | | | | | | | | | |
| --- | --- | --- | --- | --- | --- | --- | --- | --- | --- | --- | --- |
|  |  | Wild type | hR163C | hG341R | hR2163H | hN2342S | hR2454H | hR2458H | hK3452Q | hR4861H | *unc-68* null |
| Frequency | Minimum | 0.063 | 0.078 | 0.128 | 0.021 | 0.057 | 0.066 | 0.093 | 0.047 | 0.046 | 0.031 |
|  | Maximum | 3.038 | 2.318 | 1.863 | 2.254 | 2.271 | 1.645 | 1.131 | 2.121 | 3.102 | 3.069 |
|  | Range | 2.976 | 2.240 | 1.735 | 2.233 | 2.214 | 1.579 | 1.038 | 2.074 | 3.057 | 3.038 |
|  | Mean | 0.346 | 0.479 | 0.456 | 0.403 | 0.492 | 0.445 | 0.435 | 0.426 | 0.287 | 0.217 |
|  | Std. Dev | 0.100 | 0.128 | 0.084 | 0.113 | 0.130 | 0.149 | 0.096 | 0.109 | 0.275 | 0.219 |
|  | SEM | 0.001 | 0.001 | 0.001 | 0.001 | 0.001 | 0.001 | 0.001 | 0.001 | 0.004 | 0.003 |
| Wavelength:length | Minimum | 0.314 | 0.287 | 0.338 | 0.356 | 0.271 | 0.288 | 0.302 | 0.324 | 0.266 | 0.330 |
|  | Maximum | 1.813 | 1.599 | 1.674 | 1.413 | 1.855 | 2.038 | 2.051 | 1.572 | 1.884 | 2.173 |
|  | Range | 1.498 | 1.312 | 1.336 | 1.057 | 1.584 | 1.750 | 1.750 | 1.247 | 1.618 | 1.843 |
|  | Mean | 0.626 | 0.582 | 0.582 | 0.610 | 0.606 | 0.586 | 0.553 | 0.588 | 0.691 | 0.696 |
|  | Std. Dev | 0.160 | 0.085 | 0.077 | 0.093 | 0.127 | 0.118 | 0.077 | 0.087 | 0.265 | 0.246 |
|  | SEM | 0.001 | 0.000 | 0.000 | 0.001 | 0.001 | 0.001 | 0.000 | 0.000 | 0.004 | 0.003 |
| Amplitude :length | Minimum | 0.047 | 0.061 | 0.072 | 0.068 | 0.047 | 0.041 | 0.055 | 0.062 | 0.049 | 0.044 |
|  | Maximum | 0.481 | 0.507 | 0.436 | 0.462 | 0.603 | 0.477 | 0.409 | 0.452 | 0.674 | 0.763 |
|  | Range | 0.434 | 0.446 | 0.364 | 0.394 | 0.556 | 0.435 | 0.354 | 0.390 | 0.625 | 0.720 |
|  | Mean | 0.184 | 0.183 | 0.170 | 0.177 | 0.163 | 0.177 | 0.165 | 0.174 | 0.164 | 0.185 |
|  | Std. Dev | 0.061 | 0.060 | 0.051 | 0.056 | 0.051 | 0.061 | 0.043 | 0.051 | 0.064 | 0.082 |
|  | SEM | 0.000 | 0.000 | 0.000 | 0.000 | 0.000 | 0.000 | 0.000 | 0.000 | 0.001 | 0.001 |
| Old Adults | | | | | | | | | | | |
|  |  | Wild type | hR163C | hG341R | hR2163H | hN2342S | hR2454H | hR2458H | hK3452Q | hR4861H | *unc-68* null |
| Frequency | Minimum | 0.061 | 0.037 | 0.070 | 0.044 | 0.056 | 0.040 | 0.049 | 0.056 | 0.026 | 0.032 |
|  | Maximum | 2.918 | 2.895 | 3.098 | 3.067 | 3.075 | 3.096 | 2.359 | 2.700 | 3.113 | 3.119 |
|  | Range | 2.857 | 2.858 | 3.028 | 3.023 | 3.019 | 3.057 | 2.310 | 2.643 | 3.087 | 3.087 |
|  | Mean | 0.232 | 0.245 | 0.200 | 0.198 | 0.283 | 0.230 | 0.166 | 0.243 | 0.179 | 0.216 |
|  | Std. Dev | 0.122 | 0.112 | 0.154 | 0.200 | 0.137 | 0.204 | 0.177 | 0.127 | 0.324 | 0.300 |
|  | SEM | 0.001 | 0.001 | 0.002 | 0.003 | 0.001 | 0.003 | 0.004 | 0.002 | 0.006 | 0.005 |
| Wavelength:length | Minimum | 0.372 | 0.378 | 0.370 | 0.320 | 0.352 | 0.351 | 0.269 | 0.330 | -4.884 | 0.293 |
|  | Maximum | 1.661 | 1.505 | 2.071 | 1.319 | 2.041 | 1.998 | 1.277 | 1.587 | 2.183 | 1.900 |
|  | Range | 1.289 | 1.127 | 1.701 | 0.999 | 1.688 | 1.647 | 1.008 | 1.257 | 7.067 | 1.607 |
|  | Mean | 0.641 | 0.634 | 0.707 | 0.665 | 0.691 | 0.694 | 0.735 | 0.725 | 0.774 | 0.823 |
|  | Std. Dev | 0.119 | 0.104 | 0.184 | 0.130 | 0.139 | 0.155 | 0.178 | 0.172 | 0.259 | 0.260 |
|  | SEM | 0.001 | 0.001 | 0.002 | 0.001 | 0.001 | 0.002 | 0.001 | 0.001 | 0.004 | 0.004 |
| Amplitude :length | Minimum | 0.059 | 0.061 | 0.043 | 0.061 | 0.039 | 0.049 | 0.055 | 0.051 | 0.025 | 0.039 |
|  | Maximum | 0.445 | 0.488 | 0.436 | 0.456 | 0.460 | 0.482 | 0.453 | 0.482 | 0.518 | 0.560 |
|  | Range | 0.386 | 0.427 | 0.393 | 0.395 | 0.421 | 0.433 | 0.398 | 0.432 | 0.493 | 0.521 |
|  | Mean | 0.201 | 0.221 | 0.207 | 0.194 | 0.215 | 0.220 | 0.194 | 0.204 | 0.199 | 0.190 |
|  | Std. Dev | 0.068 | 0.068 | 0.076 | 0.071 | 0.074 | 0.075 | 0.078 | 0.064 | 0.082 | 0.087 |
|  | SEM | 0.000 | 0.000 | 0.001 | 0.000 | 0.000 | 0.001 | 0.001 | 0.000 | 0.001 | 0.001 |

**Supplementary table 2**. Data extracted from videos of crawling *C. elegans* for RyR variant strains, wild type and the CB540 *unc-68(e540)* null mutant. Minimum and maximum values are provided, with the range, mean standard deviation (Std. Dev) and Standard error of the mean (SEM) for Frequency, and for Wavelength and Amplitude relative to body length, for young and old adults.
